# State Medicine, Public Health, Hygiene and Bacteriology

**Published:** 1895-06

**Authors:** Ernest Wende

**Affiliations:** Health Commissioner in the City of Buffalo, N. Y.


### STATE MEDICINE, PUBLIC HEALTH, HYGIENE AND BACTERIOLOGY.

Conducted by ERNEST WENDE, M. D.,
Health Commissioner in the City of Buffalo, N. Y.

### THE MILK SUPPLY OF BUFFALO.

DEPARTMENT OF HEALTH, OFFICE HEALTH COMMISSIONER.

*Dr. Henry R. Hopkins, Chairman Sub-Committee appointed by the Academy of Medicine for Investigating the Milk Supply of Buffalo :*

My Dear Doctor—As requested by you, I herewith submit the following data concerning the milk business of Buffalo, and which would have been handed you before but for your informing Dr. Heath there was no hurry in the matter :

Annual consumption of milk in Buffalo, 5,127,660 gallons, or about 14,000 gallons per day. Of this, 550,000 gallons per annum is brought by men who haul the product from their own dairies ; 38 city milkmen keep their own herds, numbering 410 heads ; 2,500 gallons per day are delivered in bottles, the balance served from cans. The source of supply is that designated by State Board of Health, Western District of New York. It is transported to the city by the following railroad companies in the proportions named and under the following conditions :

*Delaware, Lackawanna & Western R. R.*

| Special Milk Train. | Maximum Distance Hauled. | Time Consumed. | Av'ge Amt. Hauled. |
|---|---|---|---|
| " | 60 miles. | 2 hrs., 20 min. | 2,750 gallons. |

Special cooled cars. All stations where milk is received and delivered enclosed and cooled.

*New York Central & Hudson River R. R.*

| No S. M. T. | M. D. H. | T. C. | A. A. H. |
|---|---|---|---|
| | From Lockport Branch. | | |
| By passenger trains. | 25 miles. | 1¼ to 1½ hours. | 2,000 gal. (est.) |
| | Main Line—From Batavia. | | |
| | 36 miles. | 1½ hours. | |

Milk sheds at regular stations ; terminal stations covered.

*West Shore R. R.*

| No S. M. T. | M. D. H. | T. C. | A. A. H. |
|---|---|---|---|
| | From Elba. | | |
| By reg. pass. trains. | 38 miles. | 1 hr., 22 min. | 616 gallons. |

Protection to the shipments at only two stations (Dunsville and Bowmansville) ; all other stations are exposed.

*Buffalo, Rochester & Pittsburg R. R.*

| No S. M. T | M. D. H. | T. C. | A. A. H. |
|---|---|---|---|
| | From Springville. | | |
| " | 32 miles. | 1½ hours. | 1,411 gallons. |

No protection to the milk at any point of shipment.

*Western New York & Pennsylvania R. R.*

| S. M. T. | M. D. H. | T. C. | A. A. H. |
|---|---|---|---|
| | From Hinsdale. | | |
| " | 65 miles. | 2 hrs., 50 min. | 4,000 gallons. |

No protection to shipments.

Concerning matters which bear upon the integrity of the milk supply at its source, health of herds, handling, and the like, the following appears pertinent:

*First.*—The average size of herds are from fifteen to sixty head, but I have no knowledge of the character and breed save that it is claimed that they are healthy.

*Second.*—The arrangements for cleansing the utensils, pans, cans, and the like, vary from scalding and airing to scrubbing, with and without the use of various cleansing alkaline powders, such as sal soda and gold dust. There appears to be no uniform custom in cleansing, though all have, apparently, in mind the necessity of such care and cleanliness and, in their individual methods, endeavor to be thorough, and they believe the results are satisfactory and show it.

*Third.*—The method of cleaning and removing the animal heat generally followed is that of placing the pans or cans in tubs or barrels of cold water, taken from wells, springs and running brooks and constantly and thoroughly stirring it with a stirrer for a varying period of time. The time generally allowed or given to this process varies at each place, depending upon the hour of milking and time of shipment, and varies from three-quarters to two and one-half hours. Where train time permits, longer time is expended, and *vice versa.* A few dairies use ice during the hot months to expedite the cooling; the majority do not.

The Department of Health, having no control or jurisdiction over the dairies, can only express its views upon what it would like to see done, and what would be an apparent improvement.

*First.*—A periodical examination of the cattle for tuberculosis by competent veterinary surgeons, by the tuberculine test, and also as to their general health, and particularly the condition of their udders, and that reports of such examinations should be, at stated periods, made to the health department at the city where the product is shipped to.

*Second.*—That inspections should be made of the fitness and sanitary condition of the barns, buildings and surroundings, in fact of all the premises where the herds are kept, and the milk, particular attention being given to the character of their quarters in Winter time. This should also include inquiry as to the possible source of water contamination.

*Third.*—That a more efficient and uniform method—preferably chemical—of cleansing the cans and utensils be adopted.

*Fourth.*—That the time allowed for cooling should be increased wherever possible, either by earlier milking or a better railroad schedule, or ice regularly used when the time is limited. If possible, the adoption of some more efficient method, if there be such, particularly where time is an element.

*Fifth.*—Brewers' malt is used as an article of food in about eighteen per cent. of the herds that are owned by the city milkmen or are fed in the vicinity of the city, and is used in the proportion of about twenty or thirty per cent. of their feed. The extent to which swill is used cannot be estimated, as, undoubtedly, in the Winter many farmers and dairymen use their cast-off refuse of a vegetable character in feeding their stock. The general opinion is, and correctly so, that swill is an offensive article of diet and should be stopped, and, therefore, stringent methods might be adopted for the prevention of such feeding, particularly in the vicinity of large cities.

Brewers' grain or malt, when fresh and not soured, is not objectionable as an article of diet, in fact practical men say it is a valuable food.

Concerning the transportation and facilities, it is to be stated that, while these at present are adequate, they could be improved upon as to the method of handling and carrying the milk, in the following manner, if possible :

*First.*—The arrangements should be such that the milk could be housed at the depots while awaiting transportation, in all kinds of weather, affording protection from the heat in Summer and inclement weather in Winter.

*Second.*—Those roads running special milk trains should, during the Summer months, use refrigerator cars or cars artificially cooled ; those handling milk on regular passenger trains should or might have a portion of the car so arranged that it could transport the milk in connection with ice.

The Department of Health has had some correspondence and directed its efforts in this direction, with the result of learning that, in the estimation of the companies, the haul is generally too short, or the business too small, or schedule arrangements too difficult to justify such an innovation. One railroad candidly stated that it would decline to handle any milk if it were possible to compel such conditions.

The ideal way of transporting milk is that adopted by the Delaware, Lackawanna & Western R. R. Co., at its eastern terminus, where the milk is transported under the direction of a milk company who runs its own express trains, which are iced, and at every station where the milk is received it is stored in ice houses awaiting the arrival of the train.

After reaching the City of Buffalo, the milk is handled or sold by about 350 dealers, large and small, all told, and is delivered to consumers in large and small wagons, 30 per cent. of which are uncovered, and with the exception of those used purely for hauling to and from the depot, are generally properly lettered and with their license number.

Bearing upon the maintenance of the proper condition of the milk while in the hands of the dealer in the city, the following are the most cogent facts: The methods of keeping the product cool and sweet and the cleanliness and sanitary condition of the premises and plumbing, particularly of the milk refrigerators or coolers where the milk is kept pending its distribution or while for sale.

Previous to the past year many of the milk houses in our city and nearly all or most of the apparatuses or receptacles for keeping milk were defective in cleanliness, plumbing and ventilation and in their attachments for that purpose. Since that time, however, Dr. Heath and Mr. Wilson, of this department, have inspected them and had their condition so altered as to make them, it is believed, sanitary, and protection is given against any danger from the absorption of gases from the overflow leading to the sewer.

The reinspections show that the majority of them are practical and safe and no further change in them at the present time seems pertinent, unless in the direction of elaboration.

In the accomplishment of this work 460 inspections were made that revealed the following condition of affairs which needed changing:

*First.*—The association of stable and milk house.

*Second.*—The association of privy vault and milk house—many being so bad as to necessitate their being immediately closed.

*Third.*—The absence of proper plumbing, it being mostly untrapped and unventilated.

When the reinspections are finished it is believed that the milk houses in Buffalo will be, as stated, practically safe.

In the matter of cleaning utensils and bottles used in delivery, there is the same want of unanimity of method as in the cases of the dairies, although the importance of cleanliness seems to be generally understood, and apparently the methods adopted are effectual and carefully carried out, which consist of scalding, steaming, washing with alkalies, soda, ventilating and the like. About 70 per cent. of the milk sellers merely rinse out the cans before returning them to the dairy, although it is not incumbent upon them, it being generally understood in the milk contracts that the dairymen are to keep in repair and in cleanliness the cans used in transportation. It is, in fact, a question between the buyer and the seller.

The methods adopted for preserving the milk while on the wagons in delivering during the Summer are wanting in uniformity or are absolutely insufficient. In uncovered wagons they consist merely in covering the cans with canvas, rubber or woolen covers, while on wagons that have tops little or no other protection is afforded; some use open wagons in winter and covered in Summer.

It is claimed that they deliver their product before the heat of the day and that no complaints are made and that they see no bad results, and these are apparently the facts. But very few milkmen surround their cans with ice during the extreme heat.

All wagons should be covered and it is believed that some system or device whereby the milk cans can be kept on or surrounded by ice while on the wagons, would be an improvement upon the present system, particularly if simple, within reason, and not to increase the cost of handling.

To sum up, the Department believes the most important features wherein the supply of milk to the city can best be maintained are outlined as follows:

*First.*—Veterinary and sanitary supervision at the dairies; education of the dairymen as to the importance and necessity for such, and for the various protective procedures that are desired, or the dangers from other neglect. The great importance and necessity for this work is best understood by referring to the results of examinations so far made. The amount of tuberculosis throughout the district which supplies Buffalo is not known but must exist, even though the dairymen claim their cattle are healthy and it is to their interest to have them so.

In parts of Niagara county, which fortunately do not supply us with milk, the disease is very active—probably 75 per cent. of all herds being affected. Of twenty-two samples from this region, examined by the state board of health sixteen were found tuberculous. One of the inspectors in that district told Dr. Heath, who has had some extensive conferences with him concerning the matter, that much of the disease was in the udders, and he had seen many cows from which pus came with the milk.

In connection with this matter and the state action concerning it, I have to say that the state legislature last year only appropriated $10,000 for carrying on the work of inspection of herds supplying milk, for tuberculosis, but has appropriated no money to pay for the destruction of the animals which are condemned. At the present time, all the inspectors can do is to make their recommendations, and, in most instances, all they can accomplish is isolation of the diseased from the rest of the herd. What is needed is a larger appropriation for the examination and to enable such cattle as are condemned to be destroyed at the expense of the state.

*Second.*—Improvement in the method of transportation, more particularly during the Summer months.

*Third.*—Protection from the various agencies liable to affect the milk while in the hands of the dealers, and which have been referred to ; and, furthermore, the prevention, as far as possible, of commercial fraud, such as watering, by the establishment of a corps of inspectors for the purpose, such as has been adopted in New York and Philadelphia.

The department, having no jurisdiction beyond the city limits, cannot enforce any action it may deem an improvement at the dairies. It hopes, however, by bringing the dairymen into closer connection with the department, through circulars of instruction, requests, and the like, to effect some changes, if not all, more especially in the direction of veterinary examination and cleanliness. Many questions of importance arise concerning changes which may or might be attempted in changing established procedures in the milk business, both in its transportation and delivery, and the department feels the necessity of much consideration and justification in whatever action it takes.

Concerning the relationship and connection between the dairies, the milkmen and the consumer, the department proposes to keep a register of milk dealers, showing the source of the milk, condition of herds, its character, and such other information as may be deemed pertinent for references. This register will be open for public inspection, and, to that extent, may be a guide as to the quality and character of the various milks—if they have or have not been examined for tuberculosis by a proper veterinary.

So far, it is appearing difficult to get the dairymen to respond as would be desired.

In reply to the specific inquiry made in your letter, you are respectfully informed that it is in evidence that the natural advantages belonging to the region used for grazing the herds that supply milk to Buffalo are everything that could be desired.

*Second.*—That the railroad facilities are abundant, but could be improved upon in the direction mentioned.

*Third.*—The danger from careless handling, dirty cows, and the like, have been touched upon in the foregoing, and while the present arrangements are not perfect, it is not considered that they carry with them any great degree of danger.

*Fourth.*—Abuses of milk undoubtedly exist in the way of dilution, but it is believed that it is not a practice, and that it is only resorted to irregularly and amongst the very smallest pedlars. No complaints have been received, nor is the addition of chemicals to the milk practised, so far as this department is informed. Milk has been complained of, and upon examination has been found to be contaminated by colostrum. In all these instances the matter has been corrected and its repetition guarded against.

The deductions drawn from the condition of the business, as known to the department, corresponds in every way with the experience of the state examiner, Mr. Zillig, and his assistant, who are doing excellently well, but the method of which Dr. Heath is the author is considered so helpful that the local boards of health are preparing to adopt the same at once.

In reply to your inquiry as to how the supply can be made the best in the world, you are informed that it is believed that such could not be consummated without an expenditure of money and the infringement of rights as to make such practically prohibitory.

Any further information which it is in our power to convey to you we will be glad to furnish.

Yours very respectfully,
ERNEST WENDE, *Health Commissioner.*

